# Brest Cancer Screening and Mobile Web App Intervention: Perceptions, Knowledge, and Needs among Native Women

**DOI:** 10.1093/geroni/igab046.3674

**Published:** 2021-12-17

**Authors:** Soonhee Roh, Yeon-Shim Lee

**Affiliations:** 1 University of South Dakota, Sioux Falls,, South Dakota, United States; 2 San Francisco State University, San Francisco, California, United States

## Abstract

American Indian (AI) women have the highest breast cancer mortality and lowest breast cancer screening rates in the U.S. The present study, in collaboration with the Yankton Sioux Tribe (YST) in South Dakota, sought to (1) identify the general public/professionals’ knowledge, attitudes, and needs for a mobile web app for breast cancer screening (wMammogram) intervention, and (2) inform development of the wMammogram intervention to improve Breast Cancer screening among YST women. Following a community-based participatory research approach, two focus groups were conducted in October 2020 with a total of 22 YST women aged 40-70 years, including 17 elderly women. Each group consisted of 11 community leaders, members, healthcare professionals. Qualitative analysis was conducted using grounded theory. Participants in both groups were generally favorable toward the wMammogram intervention, and noted a potential health benefit, particularly for women in their 50s to 60s. Key areas identified by participants for intervention include: (1) needs for better knowledge of breast cancer, screening, and prevention/early detection, (2) culturally tailored educational materials rooted in AI cultural values and beliefs (e.g., holistic wellness approach, Native lifestyles), (3) barriers (e.g., fear), (4) motivators (e.g., reminders), and (5) suggestions for acceptability (e.g., content and structure of messages). These results suggest that the wMammogram intervention, which is culturally tailored and addresses the community’s concerns, can be a feasible, acceptable, and effective tool to promote breast cancer screening among YST women. The results informed the development of an innovative health intervention to help reduce health disparities experienced in Indian Country.

